# Methicillin-resistant *Staphylococcus aureus* of the clonal lineage ST5-SCC*mec*II-t2460 was associated with high mortality in a Wuhan hospital

**DOI:** 10.1007/s42770-021-00557-5

**Published:** 2021-07-08

**Authors:** Xuehan Li, Jing Zhang, Yifan Zhang, Junying Zhou, Xinwei Li, Ruo Feng, Yirong Li

**Affiliations:** 1 Department of Laboratory Medicine, The Fifth Affiliated Hospital of Zhengzhou University, Zhengzhou, China; 2 Department of Laboratory Medicine, Beijing Haidian Maternal and Child Health Hospital, Beijing, China; 3 Department of Laboratory Medicine, The Affiliated Cancer Hospital of Zhengzhou University, Zhengzhou, China; 4 Department of Laboratory Medicine, Zhongnan Hospital of Wuhan University, Wuhan, China; 5 School of Basic Medical Sciences, Zhengzhou University, Zhengzhou, China; 6 Department of Histology and Embryology, School of Basic Medical Sciences, Zhengzhou University, Zhengzhou, China

**Keywords:** Methicillin-resistant *Staphylococcus aureus*, ST5-SCC*mec*II-t2460, 30-day mortality, Procalcitonin, Antimicrobial susceptibility test

## Abstract

Methicillin-resistant *Staphylococcus aureus* (MRSA) is an important human pathogen that can cause serious infectious diseases. An emerging MRSA strain, ST5-SCC*mec*II *spa*-type-t2460 (SMRSA), has spread rapidly since its recent emergence in China, but little information is available about this lineage. In this study, 91 MRSA isolates were collected from patients treated in the Zhongnan Hospital, Wuhan University, from 2018 to 2019, and investigated for their molecular characteristics, antibiotic resistance profiles, and clinical characteristics. The predominant lineage, SMRSA, accounted for 37.4% (34/91) of the isolates, followed by ST239-SCC*mec*III-t030 (19.8%, 18/91) and ST59-SCC*mec*IV-t437 (8.8%, 8/91). In contrast to the latter two non-SMRSA (nSMRSA) lineages, which are among the main MRSA found in Chinese settings, SMRSA exhibited small colony variant (SCV) phenotype and had extremely high resistance rates to erythromycin (100.0%), clindamycin (100.0%), levofloxacin (100.0%), tetracycline (97.1%), moxifloxacin (97.1%), and ciprofloxacin (100%), but was more susceptible to rifampicin (resistance rate 3%). The levels of white blood cells (WBC) and procalcitonin (PCT) and the 30-day mortality in patients infected with SMRSA were (12.54 ± 6.61) × 10^9^/L, 0.66 ng/mL, and 52.9%, respectively, which were much higher than those in patients infected with nSMRSA. In addition, patients infected with SMRSA were more frequently admitted to the intensive care unit (ICU) and submitted to invasive procedures than those infected with nSMRSA. In conclusion, SMRSA showed SCV phenotype and exhibited multiple antibiotic-resistance profiles. In this study, SMRSA was associated with serious infections and poor prognosis. Compared with ST239, ST59, or other nSMRSA strains, patients infected with SMRSA strains have higher 30-day mortality, increased levels of inflammatory biomarkers, and more frequent ICU hospitalization and invasive procedures.

## Introduction

Methicillin-resistant *Staphylococcus aureus* (MRSA) is an important human pathogen that can cause a variety of diseases ranging from mild localized infections to severe systemic infections [1–3]. Since the first detection in 1961 in Europe, MRSA isolates have become a leading cause of nosocomial infection throughout the world [4]. The prevalence of MRSA across China has declined in recent years, but it remained at 33.6% in the first half of 2020 (www.chinets.com). Recently, diverse molecular techniques have been applied to monitor the emergence of pandemic clones [5], and the molecular and epidemiological characteristics of MRSA have been investigated exhaustively in China. The molecular characteristics of MRSA strains vary with the geographic regions and have been related to complications, disease severity, and mortality [6, 7]. Since 2013, SMRSA has emerged in China and has rapidly increased in number. In 2017, SMRSA even replaced ST239-SCC*mec*III-t030 and ST5-SCC*mec*II-t002 becoming the predominant MRSA clonal lineage in Shanghai [8, 9]. Meanwhile, our previous study showed that SMRSA was the predominant clone causing bloodstream infection in Wuhan [10].

The SMRSA strains were first found in South Korea causing an outbreak in 2007 [11]. In China, the prevalence of SMRSA has importantly increased in recent years. However, little information is available about the clinical characteristics and the evolutionary process of the SMRSA clone. Moreover, although many studies have investigated the molecular characteristics of MRSA in China [8, 12–14], few have explored the relationship between these molecular characteristics and the clinical prognosis of patients, especially for the predominant MRSA lineages. In this study, we identified some clinical features of SMRSA isolates and the clinical outcomes of SMRSA-infected patients in comparison with nSMRSA infections.

## Materials and methods

### S. aureus isolates

The study was performed at Zhongnan Hospital of Wuhan University. Ninety-one non-duplicate MRSA isolates were collected from the hospital’s microbiology diagnostic lab. These isolates were derived from diverse clinical specimens of adult inpatients (age > 18 years) who had cough, fever, and other clinical symptoms related to infections and were hospitalized between January 2018 and December 2019. The specimens included skin/soft tissue (*n* = 15, 16.5%), sputum (*n* = 38, 41.8%), blood (*n* = 20, 22.0%), and others (catheter tip, drainage liquid, ascites, and bronchoalveolar lavage fluid) (*n* = 18, 19.8%).

Isolates were identified as *S. aureus* using conventional microbiological methods including Gram staining, catalase tests, and coagulase tests, and then further identified using the VITEK 2 Compact system and the VITEK 2 AST-GP67 Test Kit (bioMerieux, Inc., Durham, NC, U.S.A.). Next, MRSA isolates were recognized by their resistance to cefoxitin and confirmed by the presence of the *mecA* gene [15]. All MRSA isolates were stored at – 80 °C until further experiments. This study was approved by the Ethics Committee of Zhongnan Hospital of Wuhan University (No.2019126), which waived the requirement for informed consent because the details of patients with *S. aureus* infection were anonymized.

### Phenotypic and molecular characteristics of S. aureus

All MRSA isolates were cultured on Columbia blood-agar at 37 °C. After 24 h, the colony morphology was observed. Subsequently, a single colony was taken for chromosomal DNA extraction as described previously [10, 16]. The extracted chromosomal DNAs were stored at – 20 °C for staphylococcal protein A (*spa*) typing, multilocus sequence typing (MLST), and staphylococcus chromosomal cassette *mec* (SCC*mec*) typing according to previous studies [10, 17]. MRSA isolates that could not be classified as any known SCC*mec* type were defined as nontypable.

### Antimicrobial susceptibility testing

Antimicrobial susceptibility was determined by the VITEK 2 Compact system (bioMerieux, Inc., Durham, NC, U.S.A.). Fourteen antibiotics were tested, including cefoxitin, clindamycin, erythromycin, gentamicin, levofloxacin, linezolid, oxacillin, penicillin, rifampicin, trimethoprim/sulfamethoxazole, tetracycline, moxifloxacin, ciprofloxacin, and vancomycin. According to the results provided by VITEK 2, cefoxitin-resistant (cefoxitin minimum inhibitory concentration ≥ 8 μg/mL) and *mecA*-positive isolates were identified as MRSA. Isolates resistant to three or more antimicrobial classes were defined as multidrug-resistant *S. aureus*. ATCC 29,213 was used as the quality control organism, and the results were interpreted according to Clinical and Laboratory Standards Institute (CLSI) guidelines (CLSI M100-S29)[18].

### Clinical data collection

We collected the following data retrospectively from the patients’ hospital medical records: age, sex, underlying disease (the most common conditions were diabetes mellitus, malignancy, chronic renal disease, chronic pulmonary disease, neurologic, cardiovascular or hepatic disease), blood indicators, the levels of procalcitonin, outcome after 30 days of infection (survival or death), source of infection (skin/soft tissue, blood, sputum, and other), and invasive procedures (trachea cannula, implantable venous access device, and catheter) performed within 1 month before infection. Based on medical records, the 91 MRSA isolates were categorized as community-acquired (CA)- or hospital-acquired (HA)-MRSA according to the previous definition [19]. CA-MRSA refers to strains that were isolated within 48 h after hospital admission from an outpatient or an inpatient who had no medical history of MRSA infection, surgery, insertion of indwelling devices, or other risk factors for HA-MRSA infection in the past year. HA-MRSA refers to strains obtained from patients who had been hospitalized for 48 h or longer.

### Statistical analysis

Statistical analyses were performed using SPSS 22.0 (IBM, Armonk, NY, U.S.A.). Categorical variables were analyzed by univariate analysis using the chi-square or Fisher’s exact tests. Continuous variables were analyzed by Student’s *t*-test. All statistical tests were two-tailed.

## Results

### Phenotypic and molecular characteristics of S. aureus

As shown in Table 1, the MRSA strains were assigned into 15 sequence types, 23 *spa* types, and 5 SCC*mec* types. Of them, 4 isolates were classified as nontypable for SCC*mec* typing. Among the 91 MRSA isolates, SMRSA accounted for 37.4% (34/91), being the predominant clone, followed by ST239-SCC*mec*III-t030 (19.8%, 18/91) and ST59-SCC*mec*IV-t437 (8.8%, 8/91), as determined by *spa* typing, MLST, and SCC*mec* typing.

**Table 1:** Molecular characteristics of 91 MRSA isolates

MLST (n)	SCC*mec* (n)	*spa* (n)
ST5(39)	II(34)	t2460(34)
	II(4)	t311(4)
	II(1)	t002(1)
ST239(24)	III(18), NT(1)	t030(19)
	III(4)	t459(4)
	III(1)	t1510(1)
ST59(11)	IV(8), II(1)	t437(9)
	IV(1)	t172(1)
	IV(1)	t441(1)
ST398(3)	NT(1)	t034(1)
	V(1)	t5435(1)
	V(1)	t5462(1)
ST764(3)	IV(1)	t002(1)
	II(1)	t601(1)
	II(1)	t1084(1)
ST45(2)	IV(1)	t116(1)
	IV(1)	t1510(1)
ST6(1)	NT(1)	t701(1)
ST22(1)	V(1)	t309(1)
ST72(1)	IV(1)	t664(1)
ST88(1)	II(1)	t3622(1)
ST121(1)	I(1)	t2091(1)
ST338(1)	V(1)	t437(1)
ST630(1)	V(1)	t4549(1)
ST845(1)	III(1)	t084(1)
ST965(1)	NT(1)	t062(1)

*MLST*, multilocus sequence typing; *spa*, staphylococcal protein A; SCC*mec*, staphylococcus chromosomal cassette *mec; n*, number of isolates in each type; *NT*, non-typeable

After 24 h, the 34 SMRSA strains all showed a similar phenotype characterized by small colony size, absence of pigmentation, and weak hemolytic activity on Columbia blood-agar. We speculated that the SMRSA strains were SCVs of *S. aureus.* We further isolated and subcultured some SCVs and confirmed that they were *S. aureus* using the VITEK 2 Compact system.

### Antimicrobial susceptibility

The 91 MRSA isolates were tested for antimicrobial susceptibility. All strains were resistant to oxacillin, cefoxitin, and penicillin. No MRSA isolate was resistant to vancomycin or linezolid. The results of antimicrobial susceptibility tests for the other nine antibiotics are shown in Table 2. Most of the MRSA strains exhibited multiple antibiotic-resistance profiles. Specifically, 89.0% were multidrug-resistant and more than 50% were resistant to seven of the nine remaining antibiotics, namely erythromycin (82.4%), clindamycin (81.3%), ciprofloxacin (75.8%), levofloxacin (74.7%), moxifloxacin (73.6%), tetracycline (67.0%), and gentamicin (56.0%).

**Table 2:** Antimicrobial resistance profiles of ST5-SCC*mec*II- t2460MRSA and non-ST5-SCC*mec*II-t2460 MRSA

Antibiotics^a^	SMRSA^b^ (*n* = 34); n(%)	nSMRSA^b^ (*n* = 57); n(%)	ST239 and ST59^b^ (*n* = 35); n(%)	Total (*n* = 91); n(%)	*p*^**c**^	*p*^**d**^
ERY	34(100.0)	41(71.9)	25(71.4)	75(82.4)	0.001	0.002
CLI	34(100.0)	40(70.2)	25(71.4)	74(81.3)	< 0.001	0.002
LEV	34(100.0)	34(59.6)	25(71.4)	68(74.7)	< 0.001	0.002
TET	33(97.1)	28(49.1)	20(57.1)	61(67.0)	< 0.001	< 0.001
RIF	1(2.9)	27(47.4)	24(68.6)	28(30.8)	< 0.001	< 0.001
GEN	20(58.8)	31(54.4)	24(68.6)	51(56.0)	0.680	0.400
MFX	33(97.1)	34(59.6)	25(71.4)	67(73.6)	< 0.001	0.004
CPFX	34(100.0)	35(61.4)	26(74.3)	69(75.8)	< 0.001	0.005
SXT	0(0.0)	1(1.8)	0(0.0)	1(1.1)	1.000	1.000
MDR	34(100.0)	47(82.5)	31(88.6)	81(89.0)	0.025	0.130

^a^ *ERY*, erythromycin; *CLI*, clindamycin; *LEV*, levofloxacin; *TET*, tetracycline; *RIF*, rifampicin; *GEN*, gentamicin; *MFX*, moxifloxacin; *CPFX*, ciprofloxacin; *SXT*, trimethoprim/sulfamethoxazole; *MDR*, multidrug-resistant

^b^ n, number of isolates in each type

^**c**^ *p*, *p* value. The resistance rate to antibiotics in SMRSA isolates was compared with those in nSMRSA

^d^ *p*, *p* value. The resistance rate to antibiotics in SMRSA isolates was compared with those in ST239 and ST59

Compared with nSMRSA, SMRSA was more resistant to erythromycin, clindamycin, levofloxacin, tetracycline, moxifloxacin, and ciprofloxacin, but more susceptible to rifampicin. In addition, the SMRSA isolates had higher rates of multidrug resistance than the nSMRSA isolates. Among the nSMRSA isolates, we focused on ST239 and ST59, the major lineages found in Chinese settings. Similar to other nSMRSA strains, ST239 and ST59 were more susceptible to erythromycin, clindamycin, levofloxacin, tetracycline, moxifloxacin, and ciprofloxacin than SMRSA, but more resistant to rifampicin. However, no important difference was found in the multidrug resistance rate between SMRSA and ST239 or ST59 MRSA.

### Clinical characteristics

The mean age of the patients was 61.87 ± 15.39 (mean ± SD) years and the age range was 28 to 97 years. Seventy (76.9%) strains were from men and 21 (23.1%) were from women. Of the MRSA isolates, 94.5% (86/91) were HA-MRSA and 5.5% (5/91) were CA-MRSA. The most common underlying diseases were cerebrovascular disease (*n* = 41; 45.1%), diabetes mellitus (*n* = 18; 19.8%), chronic renal disease (*n* = 17; 18.7%), malignancy (*n* = 16; 17.6%), cardiovascular disease (*n* = 14; 15.4%), chronic pulmonary disease (*n* = 12; 13.2%), and hepatic disease (*n* = 11; 12.1%). As for the patients’ outcomes, 62 patients (68.1%) survived while the remaining 29 (31.9%) died.

As described, we also collected blood indicators, PCT levels, and information on any invasive procedures and ICU hospitalization undergone by the patients to further compare the effects of SMRSA and nSMRSA infections. The clinical information is shown in Table 3. The sex, age, underlying diseases, and sources of infection were not very different between patients with SMRSA infections and those with nSMRSA infections. However, the PCT level, WBC count, frequency of ICU hospitalization, frequency of invasive procedures within 1 month, and 30-day mortality were higher in patients with SMRSA infections than in those with nSMRSA infections. According to a comparison of the clinical features and patient outcomes between patients infected with SMRSA and those infected with the specific nSMRSA lineages that are most common in Chinese settings (ST239 and ST59), patients with SMRSA infections had higher levels of WBCs, higher 30-day mortality, more frequent ICU hospitalization, and more frequent invasive procedures than those with ST239 and ST59 infections.

**Table 3:** Analysis of risk factors for ST5-t2460-SCC*mec*II MRSA infection

	n(%)/mean ± SD/median (Q1-Q3)				*p*^**a**^	*p*^**b**^
	SMRSA (*n* = 34)	nSMRSA (*n* = 57)	ST239 and ST59 (*n* = 35)	Total (*n* = 91)		
Sex, M/F	28/6	42/15	25/10	21/70	0.342	0.282
Age, years	63.97 ± 16.48	60.61 ± 14.71	61.40 ± 14.73	61.87 ± 15.39	0.317	0.497
PCT (ng/mL)*	0.66(0.19–2.54)	0.21 (0.06–1.61)	0.41 (0.06–1.71)	0.41 (0.12–1.71)	0.027	0.146
Blood indicators*						
WBC (× 10^9^/L)	12.54 ± 6.61	9.49 ± 4.11	9.62 ± 3.96	10.63 ± 5.36	0.019	0.031
RBC (× 10^12^/L)	3.06 ± 0.71	3.25 ± 0.75	3.27 ± 0.83	3.18 ± 0.74	0.226	0.247
HGB (g/L)	91.53 ± 21.68	96.68 ± 20.84	95.67 ± 21.38	94.76 ± 21.18	0.263	0.427
PLT (× 10^9^/L)	181.00 (101.25–246.50)	196 (131.50–282.00)	194.00 (140.00–258.00)	194.00 (123.00–278.00)	0.297	0.337
NEUT (%)	85.30 (78.58–89.25)	81.50 (74.95–89.40)	83.60 (75.00–90.00)	82.80 (75.10–89.30)	0.288	0.862
LYMPH (%)	6.25 (4.58–10.30)	8.30 (5.15–17.00)	7.90 (5.00–14.10)	7.00 (5.00–12.80)	0.111	0.337
MONO (%)	7.83 (5.98–11.71)	7.40 (4.90–9.95)	6.60 (5.00–9.00)	7.50 (5.30–10.10)	0.458	0.208
Healthcare-associated, HA/CA	34/0	52/5	32/3	86/5	0.193	0.248
Underlying disease						
Cerebrovascular disease	17 (50.0)	24 (42.1)	18 (51.4)	41 (45.1)	0.464	0.906
Diabetes mellitus	7 (20.6)	11 (19.3)	10 (28.6)	18 (19.8)	0.881	0.442
Chronic renal disease	7 (20.6)	10 (17.5)	7 (20.0)	17 (18.7)	0.719	0.952
Malignancy	7 (20.6)	9 (15.8)	3 (8.6)	16 (17.6)	0.561	0.282
Cardiovascular disease	7 (20.6)	7 (12.3)	5 (14.3)	14 (15.4)	0.288	0.490
Chronic pulmonary disease	7 (20.6)	5 (8.8)	7 (20.0)	12 (13.2)	0.197	0.952
Hepatic disease	6 (17.6)	5 (8.8)	4 (11.4)	11 (12.1)	0.355	0.695
Source of infection						
Skin/soft tissue	1 (2.9)	9 (15.8)	5 (14.3)	10 (11.0)	0.121	0.213
Sputum	16 (47.1)	22 (38.6)	13 (37.1)	38 (41.8)	0.428	0.404
Blood	11 (32.4)	9 (15.8)	7 (20.0)	20 (22.0)	0.065	0.243
Other	6 (17.6)	17 (29.8)	10 (28.6)	23 (25.3)	0.196	0.282
ICU hospitalization	28 (82.4)	23 (40.4)	16 (45.7)	51 (56.0)	< 0.001	0.002
Invasive procedure	33 (97.1)	37 (64.9)	24 (68.6)	70 (76.9)	< 0.001	0.002
30-day mortality	18 (52.9)	11 (19.3)	6 (17.1)	29 (31.9)	< 0.001	0.025

*M*, male; *F*, female; *SD*, standard deviation; *PCT*, procalcitonin; *WBC*, white blood cell; *RBC*, red blood cell; *HGB*, hemoglobin; *PLT*, platelets; *NEUT*, neutrophil; *LYMPH*, lymphocyte; *MONO*, monocytes; *HA*, hospital-acquired; *CA*, community-acquired; *ICU*, intensive care unit

^*^The reference values of PCT and blood indicators: PCT < 0.05 ng/mL; WBC (3.5–9.5) × 10^9^/L; RBC (4.3–5.8) × 10^12^/L (male), (3.8–5.1) × 10^12^/L (female); HGB (130–175) g/L (male), (115–150) g/L (female); PLT (125–350) × 10^9^/L; NEUT (40–75)%; LYMPH (20–50)%; MONO (3–10)%

^**a**^The resistance rate to antibiotics in SMRSA isolates was compared with those in nSMRSA

^**b**^The resistance rate to antibiotics in SMRSA isolates was compared with those in ST239 and ST59

## Discussion

*S. aureus*, especially MRSA, has been considered a serious threat to public health for several decades [1]. The predominant MLST of MRSA in China were previously reported to be ST239 [20–23] and ST59 [12]. However, according to the results of this study, in this hospital, SMRSA was the dominant MRSA, followed by ST239 and ST59. This finding is consistent with recent epidemiological changes in MRSA in China [9, 20]. SMRSA has rapidly increased in the recent past and become the predominant clone in some parts of China [9, 20], such as Wuhan where this study was carried out. Given the rapid increase in SMRSA infection rates among inpatients, it is urgently necessary to understand the prevalence, antimicrobial susceptibility, and clinical prognosis of SMRSA infections.

As a newly emerging clone, SMRSA prevalence has increased rapidly, but so far, SMRSA has been reported only in Asia, including China, South Korea, and Malaysia [9, 10, 24–26]. This may be due to the fact that many studies do not perform *spa* typing and/or because the frequency of SMRSA is limited in other regions. In South Korea, Kim et al. have identified SCVs in two patients who presented with MRSA bacteremia, nine blood isolates were collected and all isolates belonged to the same genotype (SMRSA) [27]. Our results showed that SMRSA clone is usually an SCV. SCVs in *S. aureus* are associated with intracellular persistence and reduced antimicrobial susceptibility, which can lead to persistent and recurrent *S. aureus* infection [27, 28]. Consistent with the report from South Korea [27], all of the SMRSA strains in the present study had the characteristics of SCVs. The comparison of the antimicrobial resistance profiles of SMRSA and nSMRSA showed that the former had much wider resistance, which is consistent with a previous study [27, 29] and may provide a plausible explanation for the rapid increase of SMRSA strains in recent years. Multidrug resistance makes SMRSA strains more competitive in the hospital setting. Compared with ST239 and ST59, which were previously the predominant lineages, the multiple resistance of SMRSA to erythromycin, clindamycin, levofloxacin, tetracycline, moxifloxacin, and ciprofloxacin may be one of the reasons why SMRSA is gradually replacing ST239-MRSA. However, compared with ST239 and ST59 or with other nSMRSA strains, the resistance rate of SMRSA to rifampicin was lower. Considering that the use of rifampicin alone to treat MRSA infections can easily lead to resistance [30], it is suggested that rifampicin combined with other antibiotics should be considered when conventional antibiotics fail to treat SMRSA infections.

Although the incidence of MRSA infections has recently declined in some regions, these bacteria still pose a serious threat to public health, with high mortality rates. In our study, the 30-day mortality was 31.9% in MRSA-infected patients, which was much higher than that reported in other studies [31]. Specifically, the 30-day mortality of nSMRSA patients was similar to those observed in previous studies [31, 32], but that of SMRSA patients was 52.9%, which is much higher than that from the main lineages in Chinese settings (ST239 and ST59) and other nSMRSA strains. These results may suggest that SMRSA strains are more virulent than nSMRSA and also explain why SMRSA is gradually replacing ST239 as the predominant MRSA strain. Taking into consideration the results of previous studies of the molecular characteristics of *S. aureus* and the outcome of infections [6, 7, 33], it is reasonable to conclude that the molecular characteristics of different *S. aureus* strains may affect the outcome of infections. Specifically, we found that SMRSA, an emerging clone in China, was associated with high 30-day mortality. Moreover, some device-related infections caused by *S. aureus* SCVs have previously been reported [34, 35]. This is consistent with our finding that SMRSA infections were associated with more frequent ICU hospitalizations and invasive procedures such as venous catheterization and tracheostomy, in which SCVs might have played a role in causing device-related infections. These results seem to suggest that SMRSA infection is related to worsening of patients’ condition. In view of this, it is not difficult to understand why SMRSA strains are hospital-acquired, as described in various studies [8–10].

The PCT level and WBC count are inflammatory biomarkers that reflect the underlying biological processes as well as disease severity. For instance, the levels of PCT may rise dramatically in cases of bacterial infection, with higher values correlating with more severe infection [36, 37]. It was reported that PCT plays an important role in the inflammatory responses and histopathological changes in *S. aureus* infections. High levels of inflammatory biomarkers on admission, particularly PCT, are associated with a higher likelihood of *S. aureus* infection [38], and a higher risk of in-hospital mortality among patients [39]. In our study, patients infected with MRSA infections had higher levels of PCT. Moreover, the PCT levels and WBC counts were higher in patients with SMRSA infections than in those with nSMRSA infections. Taking into consideration the high fatality rate together with multiple antibiotic resistance of SMRSA, we conclude that SMRSA may be linked with more serious infections and poorer prognosis than other strains and we should pay more attention to the spread of SMRSA.

Our study has some limitations. First, we ran univariate statistical analysis with small sample size, which may have caused information bias. Second, the retrospective collection of data and lack of some inflammatory biomarkers, such as interleukin 6, C-reactive protein, and erythrocyte sedimentation rate, limited the informativeness. Third, the study was performed in a single center, which prevents broad representativeness. Thus, appropriate caution should be taken when interpreting our data.

## Conclusion

In this study, SMRSA was the predominant clone, followed by ST239-SCC*mec*III-t030 and ST59-SCC*mec*IV-t437. Phenotypically, SMRSA was an SCV of *S. aureus* and was associated with multiple antibiotic resistance, but was susceptible to rifampicin. SMRSA strains were also related to serious infections and poor prognosis. Patients with SMRSA had higher 30-day mortality, increased levels of inflammatory biomarkers, and more frequent ICU hospitalization and invasive procedures.

## Data Availability

The data of this study are presented in the article text and tables. Additional details are available by contacting the corresponding author upon reasonable request.
